# Enhanced Quantum Dot Light Emission at Telecom Wavelengths on Metallic Mirrors

**DOI:** 10.3390/nano16161009

**Published:** 2026-08-17

**Authors:** Ranbir Kaur, Mohanad Alkaales, Mohamed Benyoucef

**Affiliations:** Institute of Nanostructure Technologies and Analytics (INA), Center for Interdisciplinary Nanostructure Science and Technology (CINSaT), University of Kassel, Heinrich-Plett-Str. 40, 34132 Kassel, Germany

**Keywords:** quantum dots, photoluminescence, extraction efficiency, telecom wavelengths, gold mirrors

## Abstract

We demonstrate the integration of molecular beam epitaxy (MBE)-grown InAs/InP quantum dots (QDs) on gold thin films, achieving a fivefold enhancement of telecom-wavelength emission compared with QDs grown on distributed Bragg reflectors (DBRs). Micro-photoluminescence (µ-PL) spectroscopy reveals a pronounced increase in PL intensity from the Au-integrated structures, highlighting the enhanced optical response enabled by the metallic mirror effect. Reflectivity measurements exhibit a characteristic dip near the QD emission wavelength, indicating increased optical absorption and reduced reflectance, consistent with improved coupling of incident light into the fabricated structure. Power-dependent measurements demonstrate background-free exciton and biexciton emission from single QDs with resolution-limited linewidths. Polarization-dependent measurements further reveal an ultra-small excitonic fine-structure splitting, reaching values as low as ~2 μeV. Finally, statistical analysis of multiple QDs confirms the reproducibility and robustness of the observed optical properties.

## 1. Introduction

Quantum information technologies such as optical quantum computing, quantum teleportation, and advanced quantum communication protocols rely on efficient sources of highly indistinguishable single photons [1,2,3,4]. Semiconductor quantum dots (QDs) have emerged as promising sources due to their discrete energy levels and compatibility with standard semiconductor fabrication techniques [5]. Their high radiative efficiency and tunable emission wavelengths make them attractive sources for quantum applications, including on-chip integration and scalable photonic circuits. Although extensive studies have developed QDs emitting at wavelengths below 1 µm, their integration with fiber-optic systems remains limited due to the high attenuation of silica fibers in this spectral range [6,7,8]. In addition to their emission spectra, QDs offer tunable optical properties through modification of their band structure and strain engineering. Beyond spectral tuning, QDs offer controllable optical properties through engineering of their size, composition, band structure, and strain distribution. Despite these advantages, the practical performance of QD-based devices is often limited by inefficient photon extraction, which arises from the large refractive-index contrast between the semiconductor material and the surrounding medium.

To address the challenges in fiber-based quantum photonic applications, research has increasingly shifted toward QDs operating in the telecom bands, where optical transmission losses in silica fibers are minimal [9,10,11]. Telecom wavelengths not only offer strong compatibility with integrated quantum photonic platforms but are also well suited for quantum photonic circuit technologies based on silicon or SiN. Within III-V-based semiconductor heterostructures, the InAs/InP material system exhibits a lattice mismatch of approximately 3.2%, substantially smaller than the 7.2% mismatch in the InAs/GaAs system. The reduced lattice mismatch minimizes strain-induced defect formation and improves material quality, making InAs/InP QDs promising candidates for quantum light sources operating at telecom wavelengths and for long-distance quantum communication applications. At the same time, GaAs-based materials have demonstrated successful single-photon emission in the C-band, highlighting significant progress in overcoming the inherent material limitations of these systems [12,13,14].

Realizing bright, efficient, and stable quantum light sources, however, requires careful optimization of QD properties through advanced semiconductor layer engineering and integration with photonic structures such as photonic crystals [15] and distributed Bragg reflectors (DBRs) [16,17]. In recent years, we have demonstrated single-photon emission from InAs QDs embedded in an InAlGaAs matrix with a fine-structure splitting (FSS) of 20 µeV [16]. Building on this work, we achieved further FSS reduction by embedding QDs in an InP matrix and coupling them to InP-based photonic crystal microcavities, reaching FSS values as low as 5 µeV [15] and down to 2 µeV on DBR-integrated structures [17]. The DBR sample used for comparison in the present work is similar to the structure reported in Refs. [16,17], consisting of 25 pairs of InP/InAlGaAs layers designed for a center wavelength of 1.55 µm.

Among the different approaches developed to enhance the light extraction from quantum emitters, metal-based materials have gained attention due to their ability to manipulate the electromagnetic field at nanoscale levels. In particular, thin gold (Au) films are highly promising because of their broadband reflectivity spanning the near-infrared to far-infrared region. Their high reflectivity, chemical stability, resistance to oxidation, and relatively small optical skin depth make Au films attractive for optical and plasmonic devices [18,19,20,21,22]. As highly reflective metallic mirrors, planar Au thin films can modify the local optical environment of nearby emitters by reflecting photons that would otherwise propagate into the substrate, thereby reducing substrate losses and enhancing photon extraction efficiency. Moreover, their broadband optical response enables efficient light management over a wide spectral range without relying on wavelength-specific resonant nanostructures.

Building on these advantages, we present an approach using thin Au films to improve the collected photoluminescence intensity of epitaxially grown InAs QDs by MBE. Rather than relying on complex nanocavity architectures, this approach emphasizes high material quality combined with carefully engineered metal integration, a broadband reflective layer that modifies the optical environment and enhances photon extraction. In this work, we systematically investigate Au-integrated single InAs/InP QDs emitting at 1550 nm. Morphological characterization was performed using atomic force microscopy (AFM), while optical investigations, including macro- and micro-photoluminescence (PL) measurements, demonstrate a fivefold enhancement in PL intensity compared with QDs grown on distributed Bragg reflector (DBR) structures.

## 2. Materials and Methods

The samples were grown by molecular beam epitaxy (MBE) on an InP substrate. The InAs QDs were embedded between InP layers, with the QD active region grown on top of a thick InGaAs layer. The InGaAs layer served as a sacrificial layer for subsequent device fabrication. Low-density QDs were formed via the Stranski–Krastanov (SK) growth mode using a procedure similar to that described in [17], resulting in emission in the telecom C-band. For morphological characterization of the QDs, an additional uncapped QD layer was grown under identical conditions.

**Figure 1:**
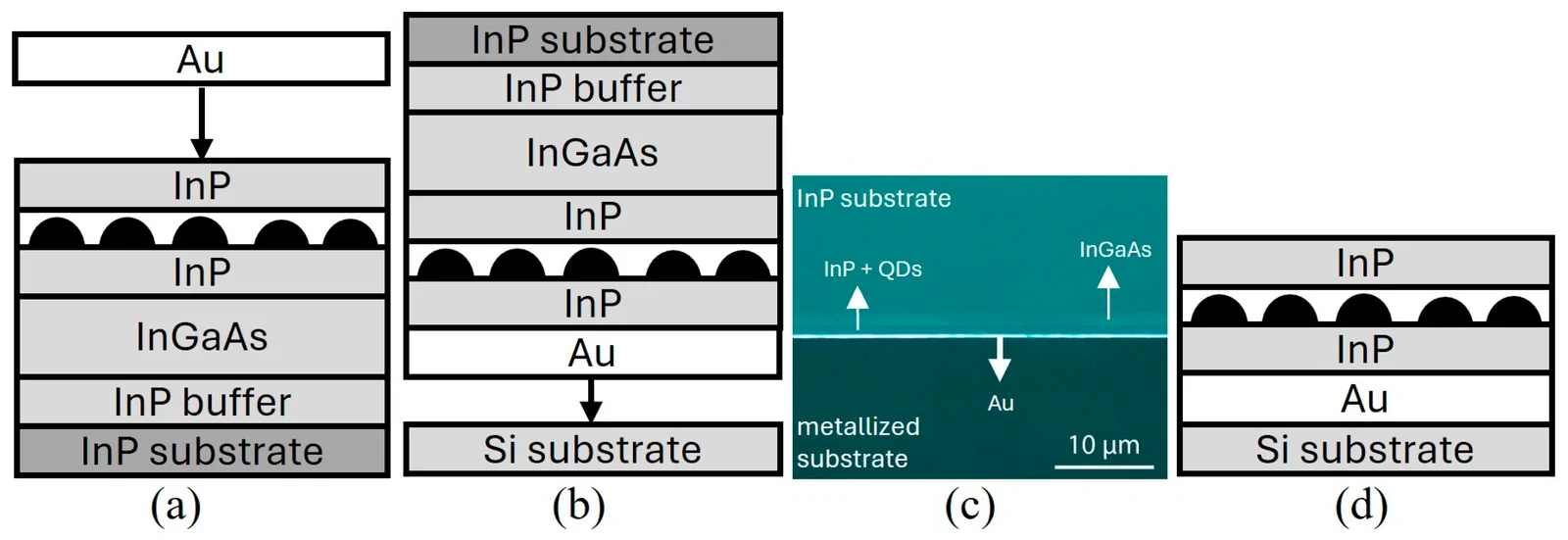
Schematic illustration of the fabrication procedure. (**a**) As-grown structure and deposition of a gold layer on the sample surface; (**b**) bonding of the gold-coated sample to a substrate; (**c**) scanning electron microscope (SEM) image of the resulting bonded structure; and (**d**) final structure, consisting of the QDs embedded between InP layers and the gold-coated semiconductor layer bonded to the substrate, used for optical characterization.

The MBE-grown samples were coated with approximately 1.0 nm of titanium (Ti), serving as an adhesion layer, followed by 200 nm of gold (Au). The separation between the QD and the Au layer is 635 nm. The Ti layer improves the adhesion between the Au film and the underlying semiconductor surface, preventing delamination [23]. After metal deposition, the sample was flip-chip bonded upside down onto a silicon substrate using conductive silver epoxy, with precise alignment achieved using a micromanipulator (Figure 1).

The heterostructure contains an InGaAs sacrificial layer that exhibits significant optical absorption near the 1.55 µm emission wavelength of the quantum dots (QDs). To enable optical access to the QDs from the substrate side, the InP substrate and the InGaAs layer were selectively removed by wet chemical etching using $HCl:H_{3}{PO}_{4}$ and $H_{3}{PO}_{4}:H_{2}O_{2}:H_{2}O$, respectively. Macro-PL measurements were performed both before and after the selective removal of the InGaAs layer, confirming the successful removal of the InGaAs layer.

**Figure 2:**
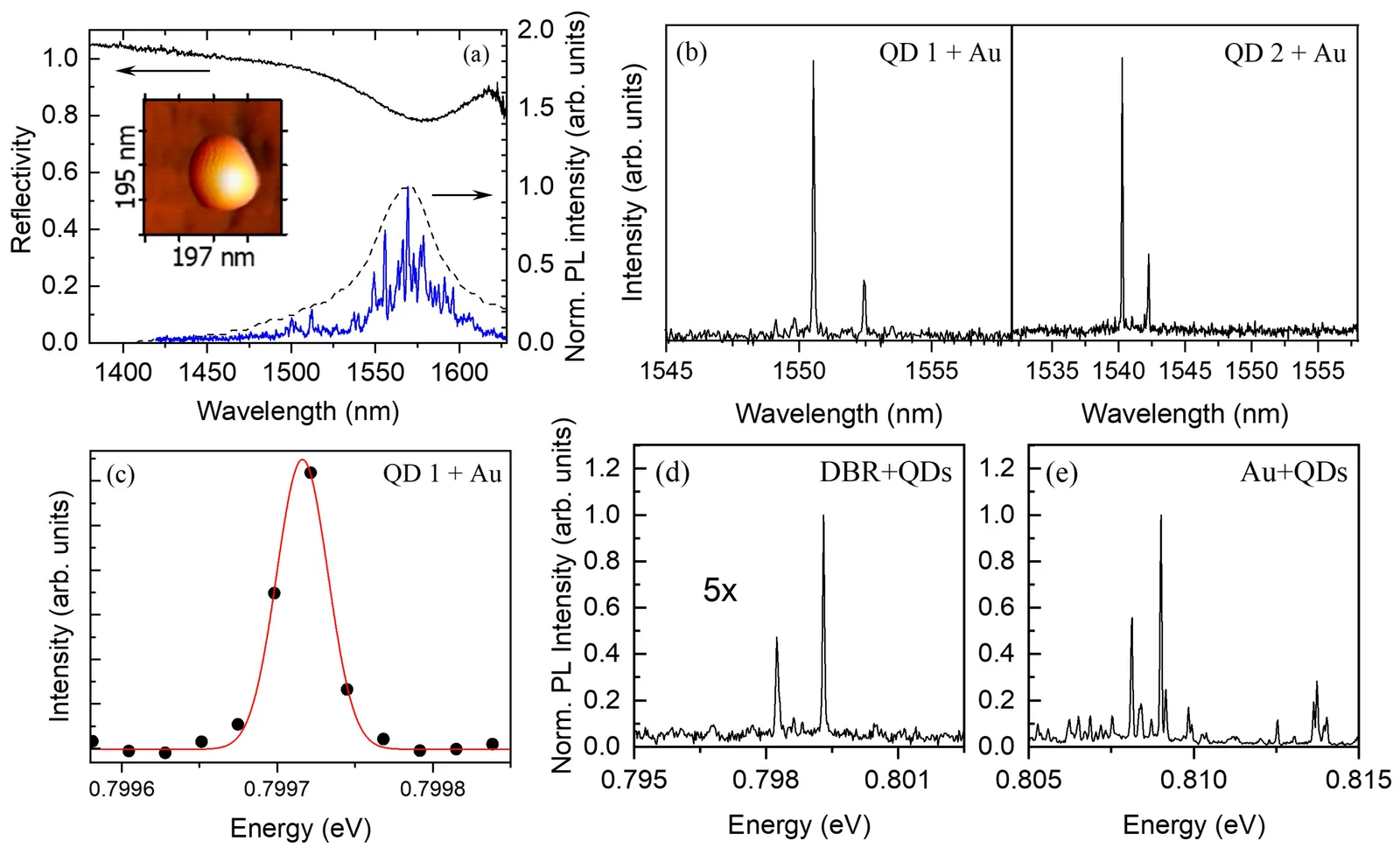
(**a**) Macro-PL (black dashed curve) and µ-PL (blue curve) spectra of the sample with a gold layer, together with the reflectivity spectrum (black solid curve). The arrow indicates the normalized PL intensities. Inset: Atomic force microscopy (AFM) image of a single QD. (**b**) µ-PL spectra of two individual QDs from the sample with the gold layer. (**c**) Resolution-limited linewidth of a single QD (QD1 + Au) emitting in the telecom C-band, the solid line represents a fit to the experimental data (dots). (**d**,**e**) Comparison of the µ-PL spectra of QDs grown on a distributed Bragg reflector (DBR) and on gold structures, measured under identical excitation conditions.

For macro-photoluminescence (PL) measurements (Figure 2a), excitation and collection were performed using a lens with a focal length of approximately 15 cm. To investigate the optical properties of individual QDs, micro-photoluminescence (µ-PL) measurements were carried out at 10 K using a helium-flow cryostat (Figure 2b–d). The sample was mounted on the cryostat cold finger and excited with a 532 nm laser focused to a spot size of approximately 1 µm through a microscope objective with a numerical aperture (NA) of 0.7. The emitted PL was collected through the same objective, dispersed by a 0.75 m spectrometer, and detected using an InGaAs detector. Polarization-resolved measurements were performed using a rotating linear polarizer.

## 3. Results and Discussion

The reflectivity spectrum of the fabricated sample was measured at room temperature, as shown in Figure 2a (black curve), revealing a reflectivity of approximately 99% in the telecom C-band, coinciding with the designed cavity resonance and the spectral region of maximum optical absorption. Macro-PL (Figure 2a, dashed black line) and µ-PL (Figure 2a, blue curve) measurements were performed at 10 K under 532 nm laser excitation. The µ-PL spectrum exhibits sharp, discrete emission lines characteristic of individual quantum dots, whereas the macro-PL spectrum is centered at the cavity resonance and closely overlaps the absorption maximum.

Background-free µ-PL spectra of individual QDs were recorded at telecom wavelengths under low excitation powers of 406 and 650 nW (Figure 2b). The full width at half maximum (FWHM) of the emission line (Figure 2c) is instrument-limited (38 µeV). These resolution-limited linewidths are consistent with the excellent optical quality of the individual QDs and suggest that their intrinsic linewidths are narrower than the instrumental resolution [24,25].

A comparative optical study was performed between QDs grown on distributed Bragg reflectors (DBRs) and QDs integrated with a thin gold layer. µ-PL spectra of both samples were recorded at 4 K under identical excitation conditions (λ_exc_ = 532 nm). Under the same excitation power of 6.4 µW, the QDs on the gold layer exhibited a fivefold increase in the measured PL intensity compared with those grown on DBRs (Figure 2d,e). The observed enhancement may be attributed to several mechanisms associated with the Au layer, including improved photon extraction resulting from optical reflection and interference, together with modifications to the local density of optical states. By contrast, plasmon-assisted coupling at the Au–semiconductor interface is expected to be negligible owing to the large separation between the emitters and the metal layer. Further studies, including time-resolved measurements, are required to distinguish the relative contributions of these mechanisms. A detailed investigation of the enhancement mechanism is currently underway and will be reported elsewhere. Notably, the emission linewidths remain resolution-limited in both structures, indicating that the gold layer does not introduce measurable spectral broadening within the resolution of our measurement system.

The observed PL enhancement may result from a combination of (i) enhanced excitation due to increased local electromagnetic fields [26], (ii) modification of the local density of optical states that alters the spontaneous emission rate, and (iii) improved photon extraction resulting from reflection by the gold layer. The relative contributions of these mechanisms remain under investigation.

**Figure 3:**
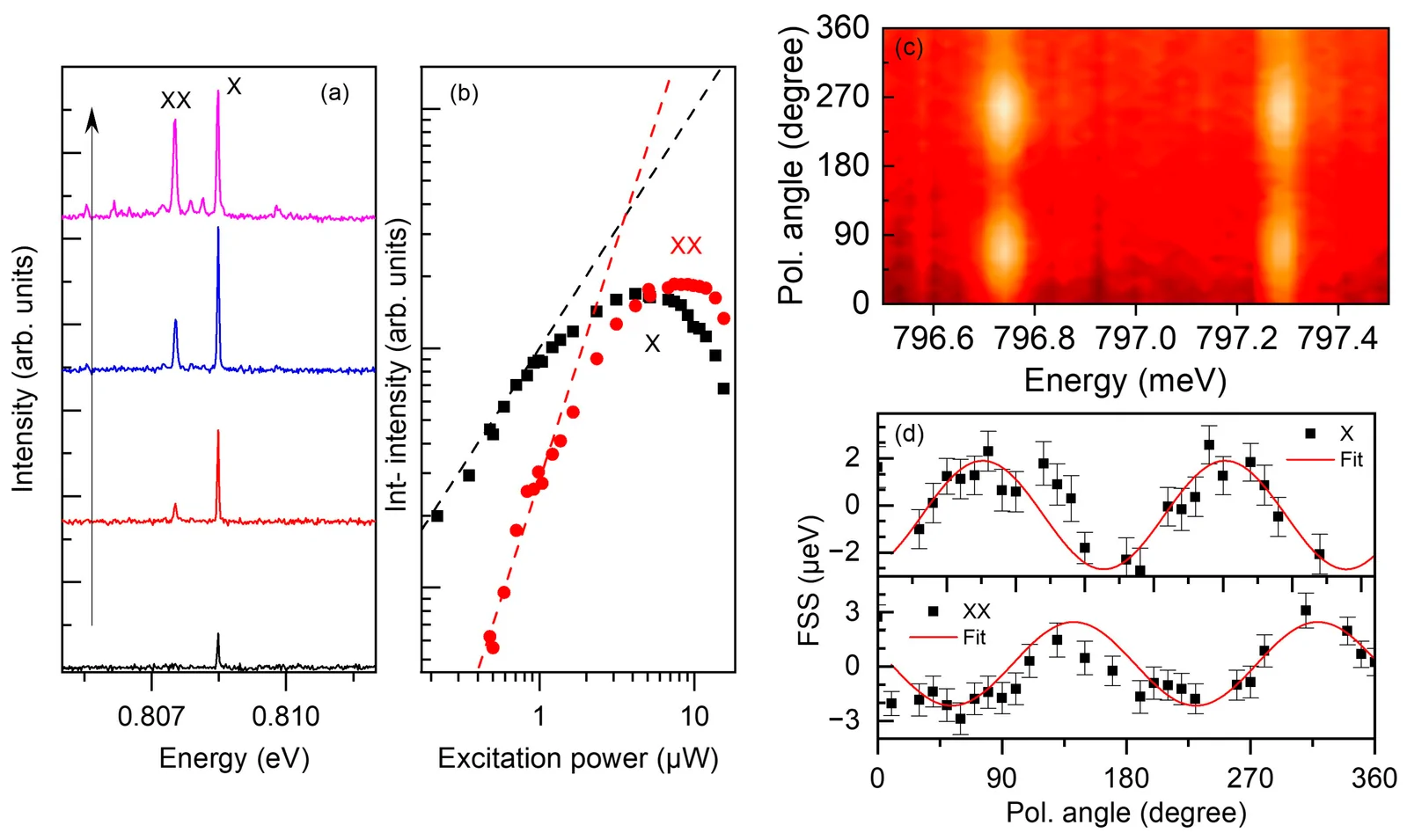
(**a**) Power-dependent µ-PL spectra of a single QD measured at increasing excitation powers, showing the exciton (X) and biexciton (XX) emission lines, the arrow indicates an increase in the excitation power. (**b**) Integrated PL intensities of the X and XX emissions as a function of excitation power, exhibiting linear and quadratic power dependences, respectively. (**c**) Polarization-dependent PL intensity map of a single QD. (**d**) Emission energies of the X and XX transitions as a function of the linear polarization angle, indicating a very small fine-structure splitting (FSS).

To investigate the excitonic dynamics, power-dependent μ-PL measurements were performed. Figure 3a shows the exciton (X) and biexciton (XX) emission intensities from a single QD as a function of excitation power. At low excitation powers, only weak exciton emission is observed owing to the low probability of carrier capture into the QD. As the excitation power increases, both the exciton and biexciton emission intensities increase significantly. The double-logarithmic plot of the μ-PL intensity versus excitation power (Figure 3b) shows that the exciton emission scales approximately linearly with excitation power, as expected for single-exciton recombination under non-resonant excitation. In contrast, the biexciton emission exhibits an approximately quadratic dependence, reflecting the sequential occupation of two excitons in the QD and becoming pronounced at higher excitation powers [15,16]. The biexciton binding energy was found to be approximately 1 meV.

Polarization-dependent μ-PL measurements were performed to investigate the fine-structure splitting (FSS) and the structural symmetry of the QDs (Figure 3c,d). The polarization-resolved energy maps (Figure 3c), plotted as a function of the linear polarization angle, exhibit the characteristic sinusoidal energy oscillations of the X and XX transitions. Fitting the polarization dependence yields a fine-structure splitting (FSS) of 2.3 μeV, indicating a high degree of in-plane structural symmetry. The small FSS, comparable to values reported for QDs grown on DBRs [15], is attributed primarily to the nearly circular in-plane geometry of the QDs, which suppresses anisotropic electron–hole exchange interactions.

**Figure 4:**
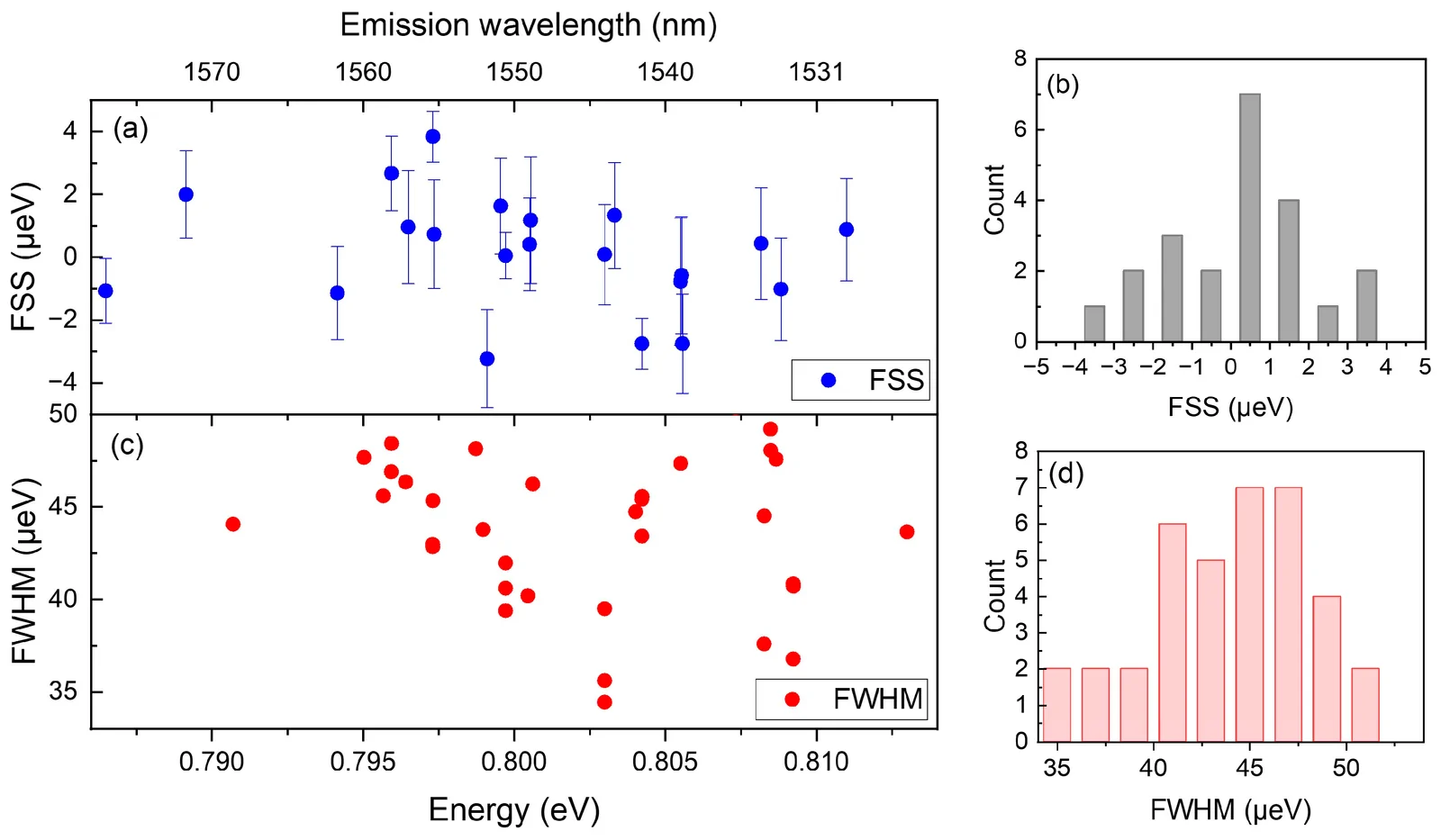
(**a**) Neutral exciton (X) fine-structure splitting (FSS) as a function of exciton emission energy for approximately 20 quantum dots (QDs) measured from the processed sample, demonstrating consistently low FSS values. (**b**) Histogram of the exciton FSS values, showing a narrow statistical distribution. (**c**) Full width at half maximum (FWHM) of the exciton emission from the same QDs, indicating narrow emission linewidths. (**d**) Histogram showing the statistical distribution of the corresponding FWHM values.

A statistical analysis of FSS and FWHM was conducted for individual QDs emitting at telecom wavelengths. As shown in Figure 4a, the FSS values remain consistently low across the emission range, consistent with a high degree of structural symmetry, an essential condition for entangled photon pair generation via the biexciton–exciton cascade [27,28].

High uniformity of the investigated QD ensemble is further supported by the distributions shown in Figure 4b. Linewidth analysis across multiple QDs (Figure 4c,d) reveals narrow emission lines of 30–35 µeV, approaching the resolution limit of our setup and thus representing an upper bound on the intrinsic linewidth. Such narrow linewidths are consistent with reduced spectral diffusion and low charge noise under the measurement conditions, reflecting excellent crystal quality.

Collectively, the combination of low FSS and narrow linewidths indicates that QDs on thin gold films are promising candidates for entangled photon generation via the biexciton–exciton cascade, which is essential for advanced quantum photonic applications.

## 4. Conclusions

We have demonstrated an efficient strategy to enhance the photon extraction efficiency of high-quality InAs/InP QDs emitting at telecom wavelengths by integrating them with thin gold films. The InAs/InP QDs on Au substrates exhibit a fivefold enhancement in telecom-wavelength emission compared with QDs integrated on DBRs. This improvement is primarily attributed to enhanced photon outcoupling and modifications of the local density of optical states induced by the Au layer. The contribution of plasmon-mediated coupling is considered negligible due to the relatively large emitter–metal separation, which limits efficient near-field interactions. Polarization- and power-dependent micro-photoluminescence measurements further confirm the high optical quality of the emitters, revealing well-resolved exciton and biexciton transitions, low fine-structure splitting, and narrow emission linewidths. These findings demonstrate that gold-integrated InAs/InP QDs provide a promising platform for bright, spectrally pure single-photon sources, offering significant potential for applications in quantum communication and integrated quantum photonic technologies.

## Data Availability

The original contributions presented in this study are included in the article. Further inquiries can be directed to the corresponding author.

## Abbreviations

The following abbreviations are used in this manuscript:

QDs	Quantum Dots
MBE	Molecular Beam Epitaxy
DBR	Distributed Bragg Reflectors
µ-PL	Micro-Photoluminescence
AFM	Atomic Force Microscopy
SEM	Scanning Electron Microscopy
FSS	Fine-Structure Splitting
